# Controlling Band-Bending for Perovskite Optoelectronic Devices Using Bismuth-Based Interlayers

**DOI:** 10.1021/acsomega.5c09745

**Published:** 2025-10-27

**Authors:** Rubén Serrano-Nieto, Wai Kin Yiu, Marcin Giza, Fraser J. Angus, Graeme Cooke, Patricia Horcajada, Yolanda Pérez, Pablo Docampo

**Affiliations:** † 202532 IMDEA Energy Institute, Advanced Porous Materials Unit (APMU), Avda. Ramón de la Sagra 3, Móstoles 28935, Madrid, Spain; ‡ School of Chemistry, 3526 University of Glasgow, Joseph Black Building, Glasgow, Scotland G12 8QQ, U.K.; § COMET-Nano Group, Departamento de Biología Y Geología, Física Y Química Inorgánica, ESCET, Universidad Rey Juan Carlos, Calle Tulipán s/n, Móstoles 28933, Madrid, Spain; ∥ Basque Centre for Materials, Nanostructures and Applications, Leioa 48940, Spain; ⊥ Departamento de Química Inorgánica, Facultad de Ciencias Químicas, Universidad Complutense de Madrid, Avenida Complutense s/n, Madrid 28040, Spain

## Abstract

Interface engineering is a powerful tool for enhancing electron/hole transfer and extraction, as well as reducing charge carrier recombination in perovskite-based optoelectronic devices, including light-emitting diodes (LEDs) and photovoltaic (PV) devices. Here, incorporating an interlayer between the perovskite and charge transport layers has been an extremely successful approach to fine-tune energy level alignment, boosting device performance. In this work, we investigate the incorporation of bismuth-based perovskitoids as interlayers to deepen the position of the perovskite’s conduction band. Our results clearly show that perovskite solar cells based on a PIN architecture with a triple-cation composition (TC) incorporating the bismuth-based interlayer outperform those without when using C_60_-fullerene (C_60_) as the electron charge extraction layer. We attribute this improvement to the deepening of the conduction band position by approximately 0.5 eV, which agrees with the X-ray photoelectron spectroscopy (XPS) and ultraviolet photoelectron spectroscopy (UPS) measurements. These findings demonstrate the potential of Bi-based perovskitoid as interlayers to induce band bending in the perovskite layer, effectively allowing fine-tuning of the energy level alignment at the device interfaces, thereby paving the way for future optoelectronic technologies.

## Graphical abstract

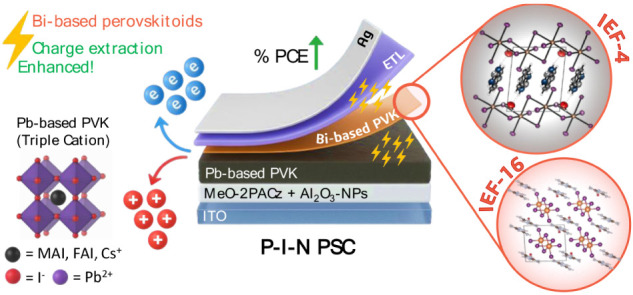

## 1 Introduction

Lead perovskite-based optoelectronic devices have garnered worldwide attention owing to (i) the excellent intrinsic photophysical properties of perovskites, including tunable bandgaps,^1^ high absorption coefficients,^2^ and efficient carrier mobility;^3^ (ii) high efficiencies (i.e., power conversion efficiency (PCE) and external quantum efficiencies (EQEs) surpassing 25% in solar cells^4^ and LEDs,^5^ respectively), (iii) their facile fabrication (i.e., low-temperature processing);^6^ and (iv) their lower production costs (i.e., simpler thin-film processes and low material usage).^7^ However, PV devices face significant challenges, such as long-term stability issues, charge accumulation, and large-scale production.^8^ On the other hand, Bi-based perovskitoids^9^ have shown promising potential due to their nontoxic nature, abundance (bismuth is a byproduct of copper, tin, and lead refining), and superior stability (toward oxygen, temperature, and moisture) compared to Pb-based counterparts.^9^ Nevertheless, their larger bandgaps (from 1.8 to 2.2 eV^10^ vs 1.47 to 2.3 eV^11^ for Bi- and Pb-based materials, respectively) and high exciton binding energies^12^ negatively impact the separation of photogenerated electron–hole pairs, limiting their potential application in highly efficient PV devices.^13^

In this scenario, interlayer engineering has shown promising results to enhance both the stability and efficiency of PV devices by improving the energy level alignment between the perovskite absorber and the charge transport layers (i.e., the electron transport layer (ETL) or the hole transport layer (HTL)), thereby promoting electron or hole extraction, respectively.^14^ A wide variety of materials, such as inorganic materials (e.g. ZnO or TiO_2_),^15,16^ organic molecules and polymers (e.g., poly­(methyl methacrylate), polyethylenimine, self-assembled monolayers),^17–20^ carbon derivatives (e.g., modified fullerenes or graphene oxide),^21–23^ or halide perovskites (e.g., PEA_2_PbI_4_)^24^ have been employed as interlayers in PV devices, achieving excellent benefits such as band alignment modulation and/or structural stability enhancement by passivation.^25^

In this arena, Bi-based perovskitoids as interlayers remain largely unexplored, with a notable exception in double perovskites such as Cs_2_AgBiBr_6_^26^ and Cs_2_BiAgI_6_,^27^ the latter using solar cell capacitance simulator software. In both cases, the double perovskite was added at the interface between the perovskite absorber layer and the HTL in a standard configuration (n-type/intrinsic/p-type or NIP), achieving promising PCEs (around 19%).^26,27^ However, no studies have been reported using an inverted configuration (p-type/intrinsic/n-type or PIN for short).

Herein, we explore, for the first time, the potential of Bi-based perovskitoid materials (i.e., IEF-4 or BzImH­[BiI_4_]·H_2_O^28^ and IEF-16 or TrzH­[BiI_4_]·H_2_O;^29^ where BzImH = benzimidazolium and TrzH = 3,5-diamino-1,2,4-triazolium) as interlayers to modulate the conduction band edge of triple-cation perovskites. We investigate the effect of these layers on standard device metrics for different PV system architectures (i.e., NIP or PIN). In this sense, manufacturing conditions were optimized by using a Bi-based interlayer to improve the Pb-based perovskite/HTL interface (NIP configuration) or Pb-based perovskite/ETL interface (PIN configuration). The results revealed that using IEF-4 or IEF-16 as an interlayer significantly deepens the conduction band edge of the Pb absorber layer, enhancing charge carrier extraction in the PIN configuration compared to the control device without an interlayer and using C_60_ as the electron extraction layer. In particular, the IEF-4-based PIN device showed an improvement in PCE, thanks to its more favorable energy level alignment with the Pb-perovskite absorber and the ETL compared to the reference device without the interlayer. Overall, we demonstrated the potential of bismuth-based interlayers to modulate energy level alignment at perovskite-based device interfaces.

## 2 Materials and Methods

### 2.1 Chemicals

All the reagents were used as received without any purification: bismuth iodide­(III) (BiI_3_, 99.9%, Alfa Aesar); bismuth­(III) oxide (Bi_2_O_3_, 99.9%, Acros Organics); aluminum oxide nanoparticles (Al_2_O_3_-NPs, 20 wt %, Sigma-Aldrich); tin­(IV) oxide nanoparticles in water (SnO_2_-NPs, 15%, Thermo Scientific); lead­(II) chloride (PbCl_2_, >99.9%, Sigma-Aldrich); lead­(II) bromide (PbBr_2_, >99.9%, Tokyo Chemical Industry); lead­(II) iodide (PbI_2_, >99.9%, Tokyo Chemical Industry); cesium­(I) iodide (CsI, >99.9%, Sigma-Aldrich); methylammonium iodide (MAI, >99% anhydrous, Sigma-Aldrich); formamidinium iodide (FAI, >99% anhydrous, Sigma-Aldrich); fullerene-C_60_ (C_60_, 98%, Sigma-Aldrich); (6,6)-phenyl C_61_ butyric acid methyl ester (PCBM, >99%, Sigma-Aldrich); bathocuproine (BCP, 96%, Sigma-Aldrich); 2-(9*H*-carbazol-9-yl)­ethyl)­phosphonic acid (2PACz, >99%, Sigma-Aldrich); 2-(3,6-dimethoxy-9H-carbazol-9-yl)­ethyl)­phosphonic acid (MeO-2PACz, >98%, Sigma-Aldrich); gold pellets (Au, 99.99%, Testbourne); silver pellets (Ag, >99.95%, Testbourne); *N*,*N*′-dimethylformamide (DMF, 99.8% Extra Dry, Thermo Scientific); diethyl ether (C_4_H_10_O, >99.7%,Sigma-Aldrich); ethyl acetate (EA, 99.9%, Thermo Scientific); isopropanol (IPA, 99.8%, Thermo Scientific); dimethyl sulfoxide (DMSO, 99.7+% Extra Dry, Thermo Scientific); chlorobenzene (PhCl, 99.8% Extra Dry, Thermo Scientific); 1,2-dichlorobenzene (PhCl_2_, 99.9%, Sigma-Aldrich); lithium bis­(trifluoromethanesulfonyl)­imide (LiTFSI, >99.9% anhydrous, Sigma-Aldrich); 2,4,7-tris­(bromomethyl)-2,1,3-benzothiadiazole (Spiro-OMeTAD, >99%, Sigma-Aldrich); 4-*tert*-butylpyridine (*t*BP, 98%, Sigma-Aldrich).

### 2.2 Preparation of the Lead-Based Perovskite Precursor Solutions

All solutions were prepared in a nitrogen-filled glovebox, stored under a dry nitrogen atmosphere, and filtered (0.45 μm, poly­(tetrafluoroethylene); PTFE) before use.

To prepare the CH_3_NH_3_PbI_3_ (MAPI) precursor solution, MAI (260.0 mg, 1.64 mmol) and PbI_2_ (750.0 mg, 1.63 mmol) were dissolved in 1 mL of a 4:1 mixture of DMF and DMSO. The precursor solution was heated at 70 °C until a clear yellow solution was obtained. To prepare the chloride-based triple cation perovskite (TC) precursor solution, CsI (19.48 mg, 0.075 mmol), FAI (208.08 mg, 1.21 mmol), MAI (33.38 mg, 0.21 mmol), PbI_2_ (726.09 mg, 1.57 mmol), and PbCl_2_ (13.90 mg, 0.05 mmol) were dissolved in 1 mL of a DMF and DMSO (4:1) mixture. The solution was heated at 70 °C overnight.

### 2.3 Synthesis of IEF-4 and IEF-16 Perovskitoids

The bismuth-based perovskitoids, IEF-4 (BzImH­[BiI_4_]·H_2_O; BzlmH = benzimidazolium)^28^ and IEF-16 (TrzH­[BiI_4_]·H_2_O; TrzH = triazolium),^29^ were synthesized following the previously reported procedures.

### 2.4 Conventional Perovskite Solar Cell Fabrication (NIP Architecture)

Patterned fluorine-doped tin oxide (FTO) glass substrates were cleaned with 8% decon-90 solution, deionized (DI) water, acetone, ethanol, and DI water. The substrates were then dried by using N_2_ and treated with UV ozone for 20 min. For the ETL, a SnO_2_ nanoparticle solution was prepared by mixing SnO_2_ nanoparticles with DI in a 1:1 volume ratio and was filtered before use; 220 μL of the ETL solution was spin-coated at 2000 rpm for 30 s. The resulting film was annealed at 150 °C for 30 min on a hot plate. The MAPI perovskite film was prepared using a dynamic spin-coating process. The procedure involved spinning at 1000 rpm for 10 s, followed by 5000 rpm for 30 s. At the 5 s mark, 50 μL of MAPI solution was deposited, followed by the addition of 300 μL of PhCl as an antisolvent, 7 s into the second step. The samples were dried on a clean cloth for 15 min and subsequently annealed at 100 °C for 15 min. The bismuth-based perovskitoid (IEF-16) was deposited on the MAPI layer. Thus, 40 μL of a solution of IEF-16 (10–15 mM) in IPA was spin-coated at 3000 rpm for 30 s and then annealed under optimized conditions (100 °C for 15 min). The HTL was formed by depositing 50 μL of a filtered Spiro-OMeTAD solution, which was prepared by dissolving 75 mg of Spiro-OMeTAD in 1 mL of PhCl and adding 10 μL of *tert*-butylpyridine (tBP) and 30 μL of Li-TFSI (170 mg·mL^–1^ in acetonitrile). The deposition was carried out by spin-coating at 1500 rpm for 40 s followed by 2000 rpm for 5 s. Finally, 40 nm of gold (Au) was thermally evaporated through a patterned shadow mask at a pressure of 10^–6^ mbar and a deposition rate of 0.1 nm·s^–1^, forming the back electrode.

### 2.5 Inverted Perovskite Solar Cell Fabrication (PIN Architecture)

The patterned indium tin oxide (ITO) substrates were cleaned and prepared using a protocol similar to that used for the standard architecture. For the HTL deposition, 3 mg·mL^–1^ of MeO-2PACz was dissolved in ethanol at 70 °C overnight. The solution was then spin-coated statically at 4000 rpm for 30 s, followed by annealing at 100 °C for 15 min on a hot plate. To enhance wettability, a solution of Al_2_O_3_ nanoparticles (using 50 μL of a 0.02%w/v solution in IPA) was deposited by spin-coating at 2000 rpm for 30 s and annealed at 100 °C for 5 min. Subsequently, 50 μL of the TC precursor solution was statically spin-coated on the self-assembled monolayer (SAM) layer at 4000 rpm for 40 s. With a delay of 15 s, 200 μL of EA as the antisolvent was quickly deposited onto the spinning film. The sample was then annealed on a hot plate at 100 °C for 30 min. A bismuth-based perovskite interlayer was deposited onto the TC layer. For this, 50 μL of a solution of IEF-4 (3.6 mM) or IEF-16 (10.8 mM) in IPA was spin-coated at 3000 rpm for 30 s and then annealed at 100 °C for 15 min. For the deposition of the ETL, a solution of PCBM in PhCl (20 mg·mL^–1^) or a solution of C_60_ in PhCl_2_ (10 mg·mL^–1^) was prepared at 70 °C overnight and filtered before use. 50 μL of the PCBM or C_60_ solution was spin-coated dynamically at 3000 rpm for 30 s and annealed at 100 °C for 15 min or 70 °C for 5 min, respectively. A hole-blocking layer was prepared by 1 mg·mL^–1^ of BCP in IPA and was spin-coated at 5000 rpm for 30 s. Finally, a 100 nm Ag layer was deposited through a patterned shadow mask by thermal evaporation at 10^–6^ mbar and a 0.5 Å·s^–1^ deposition rate to form the back electrode.

### 2.6 Characterization

X-ray diffraction (XRD) measurements were performed with a Bruker D8 Discover X-ray diffractometer operating at 40 kV and 30 mA, employing Ni-filtered Cu Kα1 radiation (λ = 1.5406 Å) and a position-sensitive LynxEye detector. The Bragg–Brentano scanning geometry or an alternative grazing-incidence geometry with an incident angle of 0.5° was applied to record the data. Powder X-ray diffraction (PXRD) patterns were recorded on an STOE powder diffractometer equipped with a position-sensitive Mythen-1K detector in transmission geometry. Scanning electron microscopy (SEM) images of pure IEF-16 films were recorded with a JEOL JSM-6500F scanning electron microscope operated at an acceleration voltage of 5 keV. SEM images of IEF-based heterostructures were collected with a TESCAN Clara S8152 instrument operated at an acceleration voltage of 15 keV, with a 300 pA beam current. Ultraviolet–visible (UV–vis) absorption spectra were recorded using a PerkinElmer Lambda 1050 spectrophotometer equipped with a 150 mm integrating sphere. Air (100% transmission) and a Spectralon white standard (100% reflection) were used as the instrument baseline. Photoluminescence (PL) spectra were acquired using an FS5 spectrofluorometer (Edinburgh Instruments) equipped with a 150 W continuous xenon arc lamp as the excitation source, Czerny–Turner monochromators (f/4.1, 1200 lines·mm^–1^), and a PMT-870 detector for the emission range 230–870 nm. Emission scans (720–870 nm, λ_exc_ = 700 nm) were collected with 3 nm slit width and 0.5 s dwell time, without polarizers. Measurements were performed under ambient conditions using the SCA-7 solid sample holder placed at 90°. Spectral correction (reference detector file) and data processing were performed via FLUORACLE (0.3 nm spectral resolution and >10^5^ stray light suppression).

Time-resolved photoluminescence measurements were conducted using an Edinburgh Instruments Time-Correlated Single Photon Counter (TCSPC) FS5 and a 508 nm laser with a 50 kHz repetition rate and a pulse fluence of ∼18.6 nJ/cm^2^.

## 3 Results and Discussion

The main goal of this study is to evaluate the potential of bismuth-based perovskitoids (i.e., IEF-4 and IEF-16) as interlayers to modulate the energy level alignment at the perovskite/charge extraction layer interface. In this sense, IEF-4 and IEF-16 were selected for their excellent optoelectronic properties, including a bandgap value of ∼2 eV and a ∼0.5 eV offset position of energy bands compared to lead-based perovskites (conduction band, CB ∼ −4.5 and ∼ −4 eV for bismuth-based perovskitoids and lead-based perovskites (PVK), respectively; Figure S1).

In order to ensure their application as interlayer materials in optoelectronic devices, we developed a deposition method that does not disrupt the underlying perovskite material. Here, IPA was selected as the appropriate solvent, avoiding dissolution of the Pb-based active layer. Trial samples deposited on glass, as shown in Figure S2, allowed fine-tuning of the deposition conditions and demonstrated that the obtained films display a similar pattern to the bulk IEF-16, confirming the film crystallization. SEM analysis of the films (Figure S3) revealed that the best film formation condition was achieved by using annealing at 100 °C for 15 min. This condition yields a uniform film with excellent crystallinity, without significant observable defects, such as pinholes or incomplete surface coverage.

Critically, the approach developed here does not disrupt the underlying perovskite absorber, as demonstrated by XRD analysis (Figure 1a). Thus, we optimized the perovskite/IEF-16 heterostructure formation by modifying the initial concentration (10–15 mM) and spin-coating parameters (1–3 k rpm for 30 s). The XRD patterns of the resulting samples showed weak reflections around 8.5°, 9.5°, and 12.6° 2θ (shifted from 12.8°), which correspond to the most intense reflections of IEF-16 (Figure 1a inset). This indicates the successful crystallization of the bismuth-based layer on top of the lead-based absorber layer, further confirmed by SEM. Figure 1b shows the initial polycrystalline MAPI perovskite. Upon IEF-16 coating, the MAPI surface is completely covered with an additional layer of small crystals. Here, the grain size is much smaller than in thin films deposited on glass (Figure S3), suggesting that the underlying perovskite greatly increases the crystallization speed.

**Figure 1:**
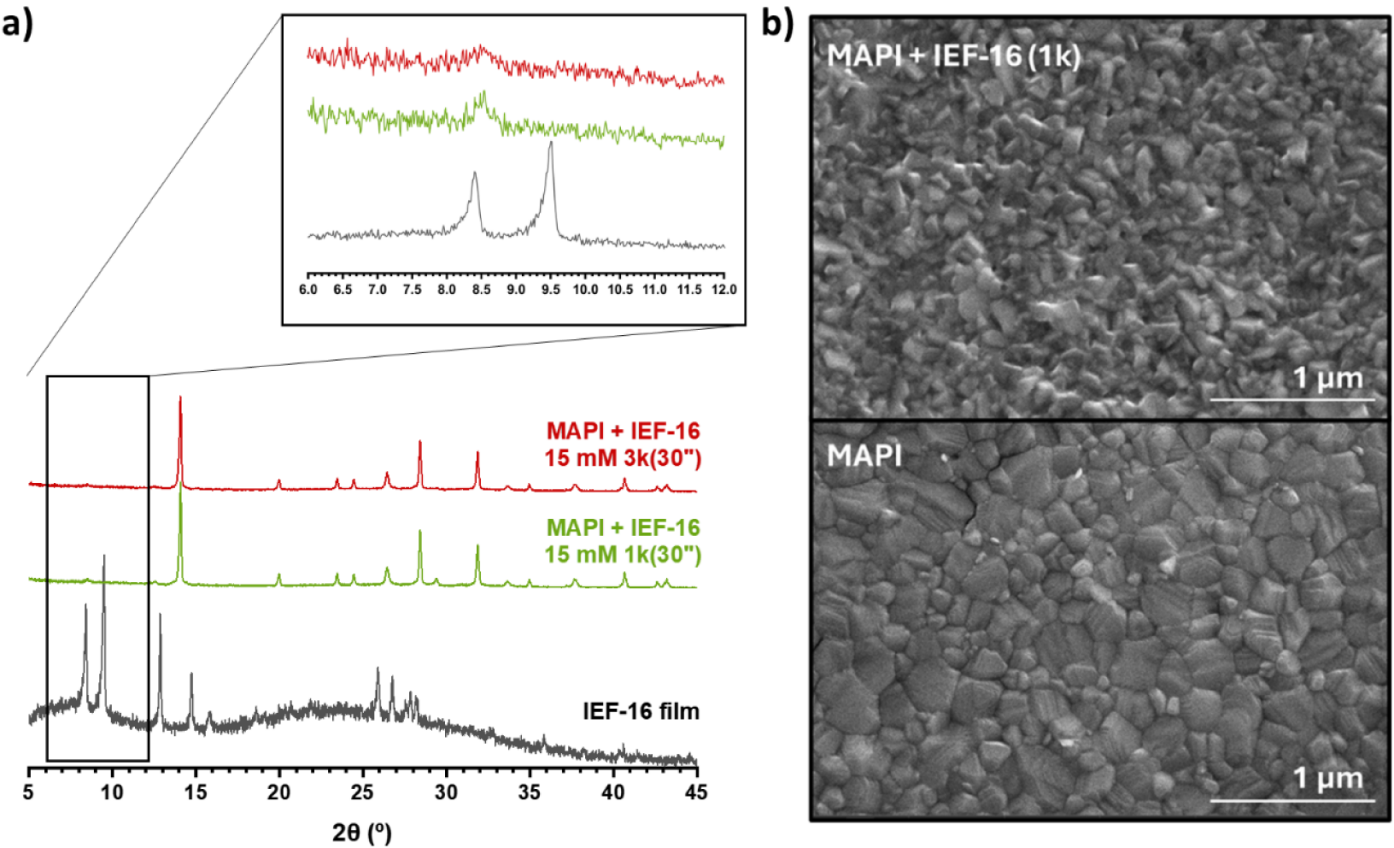
a) XRD patterns of the IEF-16 film and the resulting films depositing IEF-16 onto the MAPI layer (MAPI + IEF-16) and b) SEM images of a MAPI thin film and one with 15 mM of IEF-16 deposited on top.

To further prove the effectiveness of the interlayer in modifying the energy-level alignment at the perovskite absorber interface, we fabricated perovskite solar cells in both PIN and NIP configurations. Here, preliminary results were obtained employing MAPI as the perovskite absorber (Figure 2), and a more optimized triple-cation composition was employed to ensure that the results are relevant to state-of-the-art device configurations.

**Figure 2:**
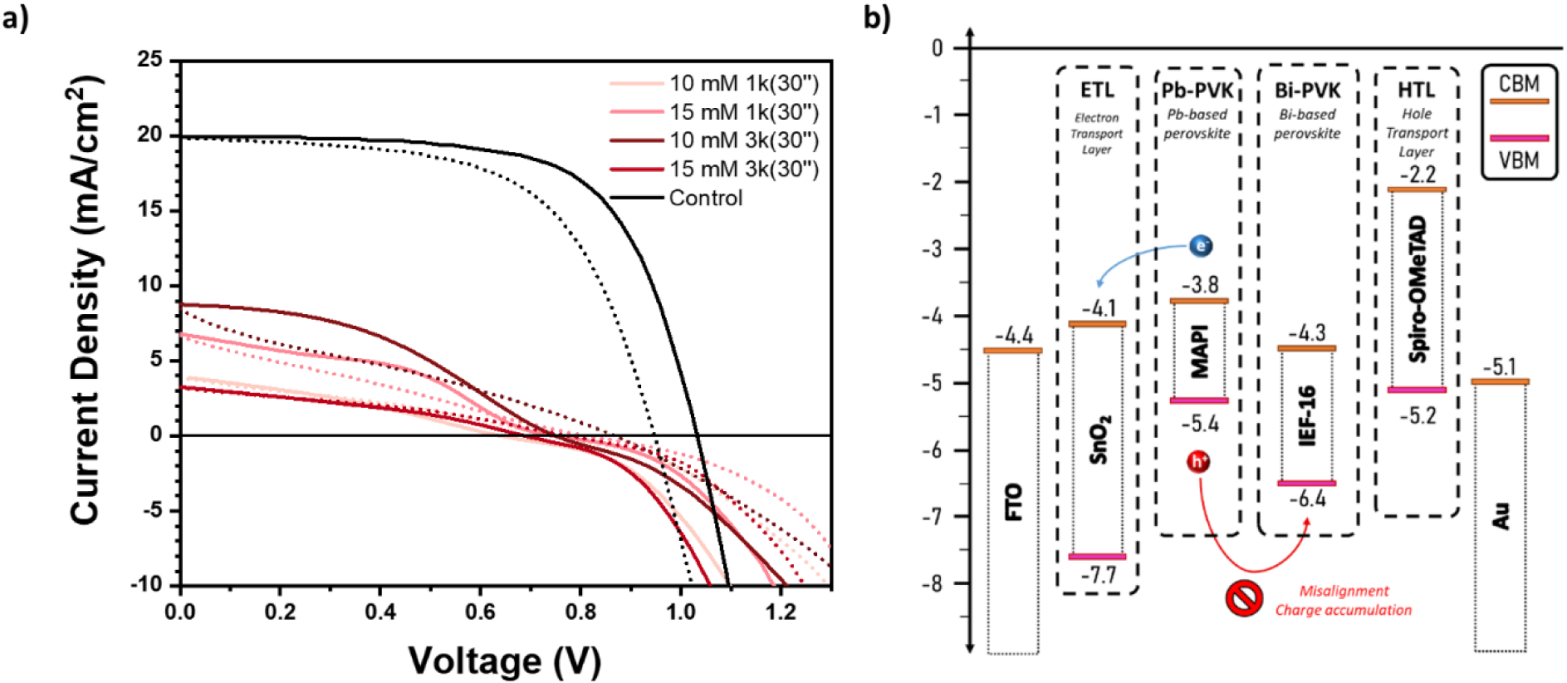
a) *J*–*V* curve and b) energy scheme of IEF-16 deposited at different concentrations as an interlayer in an NIP configuration of the perovskite-based solar cell (FTO/SnO_2_/MAPI/IEF-16/Spiro/Au).

NIP devices were fabricated with SnO_2_ as the ETL, MAPI as the absorber layer (intrinsic material), IEF-16 as an interlayer, Spiro-OMeTAD as the HTL, and gold as the contact electrode (i.e., FTO/SnO_2_/MAPI/Interlayer/Spiro-OMeTAD/Gold). The bismuth interlayer was deposited from a range of concentrations (10–15 mM) and spin-coating protocols (1–3 k rpm for 30 s), with some representative devices displayed in Figure 2a. As can be seen in the *J*–*V* curves, there is a clear reduction in all photovoltaic parameters concerning the reference sample without the bismuth-based interlayer. Notably, all devices incorporating the interlayer exhibited a pronounced S-shape, which is a phenomenon often associated with charge accumulation and inefficient charge extraction due to poor band alignment between the layers.^30^ These results indicate that the band bending introduced by the bismuth interlayer led to a significant mismatch among the energy levels of MAPI, IEF-16, and Spiro-OMeTAD (−5.4, −3.9, and −5.2 eV, respectively). This then leads to a clear impediment to efficient charge extraction, creating a significant barrier for hole transfer (Figure 2b) and likely increased recombination, with a subsequent loss in power conversion efficiency (PCE < 3%, Figure S4).

Although the S-shape indicates the successful modulation of the underlying perovskite’s conduction band edge, it is important to note here that this phenomenon can also arise upon introducing interfacial defects.^31,32^ In order to conclusively prove this, we additionally fabricated devices in an inverted configuration (PIN) employing standard materials for this device architecture: ITO/MeO-2PACz/Al_2_O_3_–NPs/TC/IEF-X/ETL/BCP/Ag. Here, BCP is a hole-blocking material used typically in inverted perovskite solar cells.^33^ For the ETL, we utilized PCBM and C_60_, which are state-of-the-art electron transport materials (ETMs) in PIN perovskite solar cells. Thus, these materials were selected to discriminate between the charge extraction kinetics, as their LUMO levels (i.e., −3.9 vs −4.5 eV for PCBM and C_60_, respectively) differ by a similar offset as the TC/IEF-X interlayer offset (i.e., 0.6–0.3 eV offset). In addition, they are well-known for their compatibility with perovskite materials and their favorable properties (e.g., electron transport properties, charge extraction, capacity to form uniform films, and passivate trap states at grain boundaries).^34–36^ To enhance charge transport efficiency, we implemented two Bi-based perovskitoids (i.e., IEF-16 and IEF-4), acting as interlayers between the perovskite layer and ETL. These materials present distinct energy levels (i.e., CB and valence band; VB, −4.6 and −4.3 eV, and −6.6 and −6.4 eV for IEF-16 ^29^ and IEF-4, respectively; Figure S1) and contain different organic cations (i.e., triazolium and benzimidazolium for IEF-16 and IEF-4, respectively).

Figure 3 shows the *J*–*V* characteristic curves of PIN devices using PCBM and C_60_ as ETLs, with their photovoltaic performance summarized in Table 1, and the whole data set is included for completeness in the (Figures S5–S8). In PCBM-based devices, the control device exhibits a PCE of 16.68%, with a short-circuit current density (*J*_sc_) of 21.14 mA·cm^–2^, an open-circuit voltage (*V*_oc_) of 0.73 V, and a fill factor (FF) of 0.73. However, the photovoltaic performance decreased when interlayer materials were introduced. The PCE dropped to 1.18 and 8.75% (for IEF-16 and IEF-4, respectively), with corresponding declines in *J*_sc_ (i.e., 2.65 and 18.40 mA·cm^–2^ for IEF-16 and IEF-4, respectively) and FF (i.e., 0.49 and 0.50 for IEF-16 and IEF-4, respectively). Furthermore, *V*_oc_ was slightly reduced to 0.90 and 0.96 V for IEF-16 and IEF-4, respectively. A detailed statistical analysis is presented in Figure S9.

**Figure 3:**
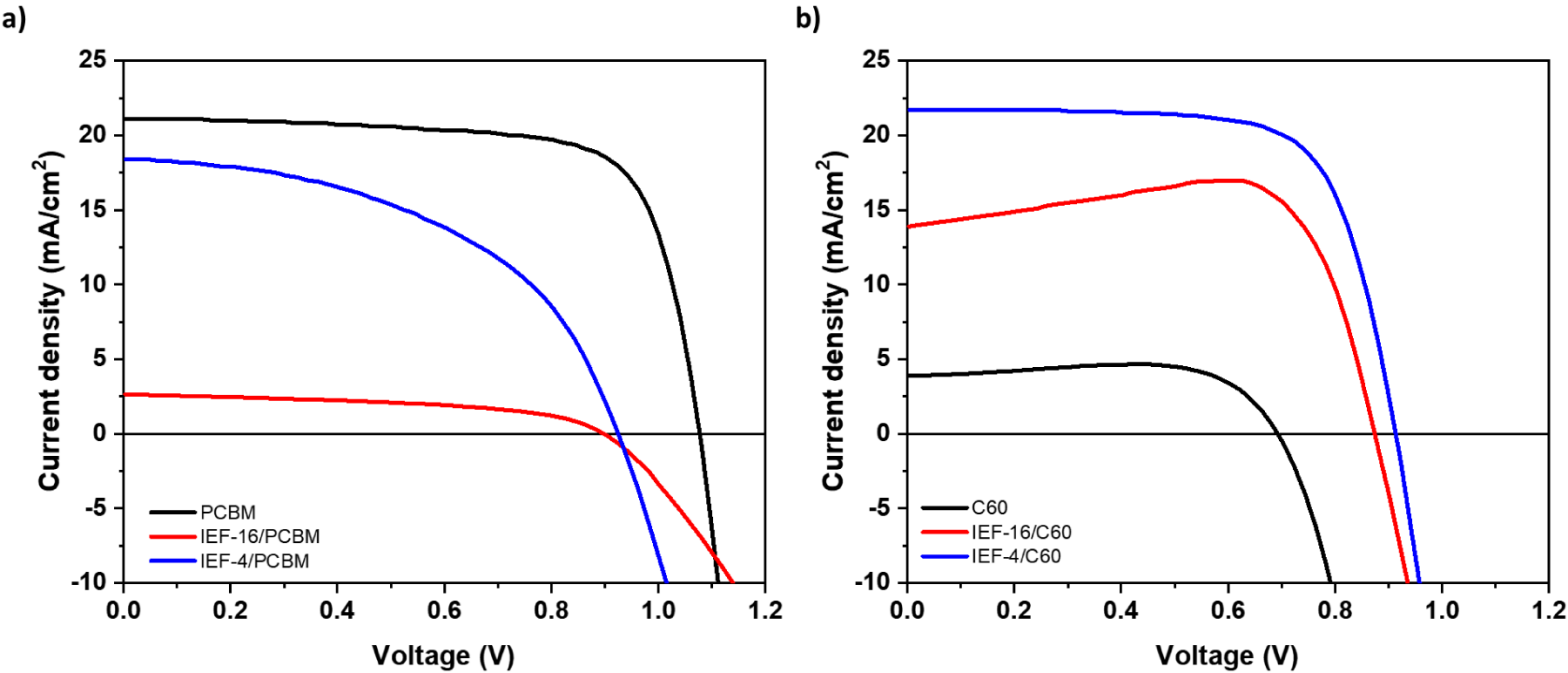
*J*–*V* curves of perovskite solar cells containing the IEF-16 or IEF-4 interlayer, TC absorber layer, and (a) PCBM or (b) C_60_ as the ETL.

**Table 1:** Photovoltaic Performance Parameters for the Control Device and the Devices Using C_60_

Absorber	Interlayer	Conc. mM	ETL	*J*_sc_ (mA·cm^–2^)	*V*_oc_ (V)	FF	PCE (%)
TC	None	-	C_60_	3.88	0.69	0.85	2.29
TC	IEF-16	10.8	C_60_	13.95	0.87	0.90	10.96
TC	IEF-4	3.6	C_60_	21.68	0.91	0.72	14.20
TC	None	-	PCBM	21.14	1.08	0.73	16.68
TC	IEF-16	10.8	PCBM	2.65	0.90	0.49	1.18
TC	IEF-4	3.6	PCBM	18.40	0.96	0.50	8.75

This decline in performance is attributed to the energy misalignment of Bi-based perovskite interlayers between the TC layer and PCBM, as illustrated in Figure 4. The CB and LUMO levels of TC and PCBM are −4.0 and −3.9 eV, respectively. Since the CB levels of IEF-16 and IEF-4 are deeper (i.e., −4.3 and −4.6 eV for IEF-16 and IEF-4, respectively), charge transfer to PCBM becomes hindered, and thus *J*_sc_ is reduced. The performance decay is more significant for IEF-16, which forms a distinct layer over the underlying perovskite (Figure S11), unlike IEF-4, which is too dilute to coat the triple-cation perovskite with a distinct layer of material. Additionally, the FF reductions stem from increased series resistance, as indicated by the slopes of the *J*–*V* curves in Figure 3.

**Figure 4:**
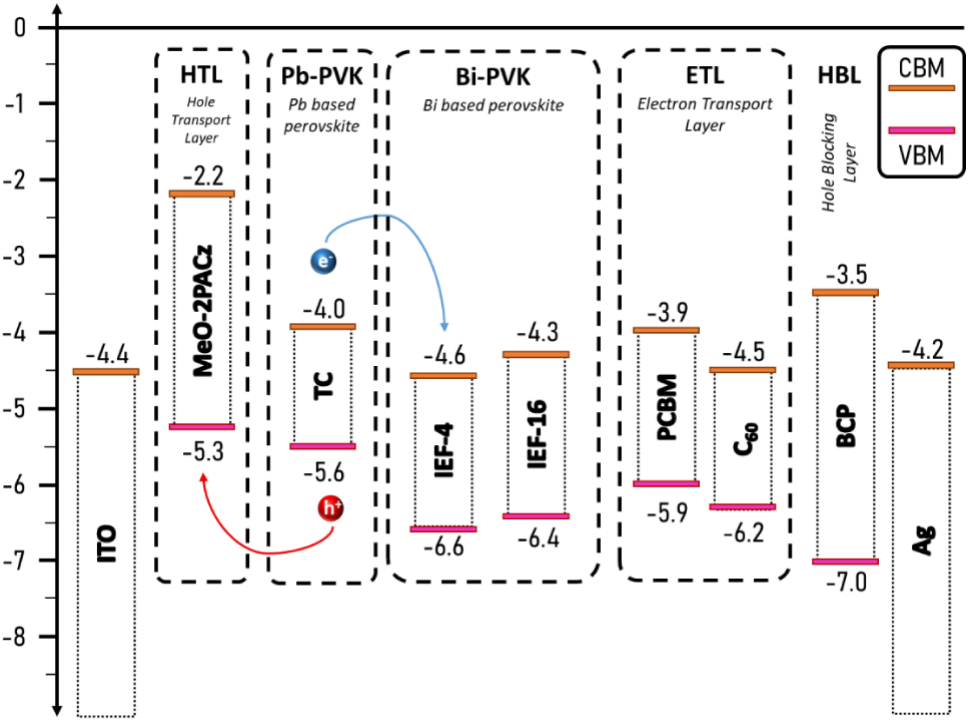
Energy scheme of a PIN architecture employing IEF-4 or IEF-16 as an interlayer and PCBM or C_60_ as the ETL.

We propose that the presence of IEF-16 crystals on the perovskite–perovskite surface can disrupt the interface between the triple-cation absorber and the ETL, leading to a reduction in FF. Therefore, C_60_ with a LUMO level deeper than that of PCBM was used as the ETL to optimize performance. However, the lower performance of the reference C_60_-based PSCs is attributed to the fabrication process, as C_60_ was dissolved in PhCl_2_ and an organic solvent in which C_60_ has relatively poor solubility compared to PCBM. This resulted in film aggregation and poor film formation.

Despite this, the devices showed notable improvements after incorporation of the Bi-based interlayers. In this sense, the PCE increased from 2.29 to 10.96 and 14.20% (for control, IEF-16 and IEF-4, respectively), primarily due to enhancements in *J*_sc_ and *V*_oc_. The *J*_sc_ rose from 3.88 to 13.95 and 21.68 mA·cm^–2^ (for the control, IEF-16, and IEF-4, respectively), attributed to better energetic alignment between the perovskite layer and C_60_ (Figure 4) and efficient charge recombination suppression according to PL measurements (Figure S7). IEF-4 outperformed IEF-16 due to its smaller energy offset with C_60_ (i.e., 0.1 vs 0.2 for IEF-4 and IEF-16, respectively). This small energetic offset facilitates more efficient charge transport by minimizing charge accumulation at the interface and reducing recombination, while still maintaining a sufficiently high built-in potential to support the high Voc in these systems.^37–39^

Meanwhile, thicker layers of IEF-16 may contribute to increased charge recombination, as shown by the statistical analysis in Figure S10. FF reductions persist after interlayer deposition, likely due to the significant accumulation of IEF-16 at the interface, which creates additional shunt pathways within the device, similar to the behavior observed with PCBM. Comparing the PCE achieved with our device to other reported devices containing Pb and Bi, the [NH_2_CHNH_2_(K_0.25_Bi_0.25_)­Pb_0.5_I_3_]-based device reached a PCE of 0.52%,^40^ the MABi_0.04_Pb_0.96_ I_3_-based device reached a PCE of 4.32%,^41^ and the Cs_3_Bi_2_I_9_/MAPbI_3_-based device reached a PCE of 11.9%.^42^ While architectures vary, these results highlight the potential of Bi perovskitoids as interfacial modifiers to adjust the band edge position and thus retain efficient device performance while preserving the optoelectronic quality of the lead-based active layer.

It is important to note here that the optimum performance for the PIN devices, including the bismuth interlayer, was achieved by employing a very dilute concentration of its casting solution, i.e., extremely thin interlayers. This is analogous to studies carried out using layered perovskites, for instance, employing phenethylammonium iodide (PEAI), where optimum performance is achieved with concentrations in the <5 mM range.^43,44^ Indeed, XRD shows that at these concentrations, the peaks corresponding to the bismuth structures are either barely visible or lost within the noise of the signal (Figure S8). Nevertheless, the effect on the device performance is still significant, indicating that even at these reduced thicknesses, there is enough material to significantly modulate the conduction band edge of the perovskite absorber. We propose that avoiding the formation of significant quantities of crystalline bismuth perovskitoids allows it to modulate the energetics of the perovskite interface without disrupting its quality or introducing additional defects.

To further substantiate this point, time-resolved photoluminescence (TRPL) measurements were conducted on samples with a configuration of Glass|PVK|IEF|ETL, where the IEF is either IEF-4 or IEF-16 and the ETL is PCBM or C_60_. The results are displayed in Figure 5a with insets to show early time dynamics. Information regarding the measurement can be found in Section 2. The PL decay clearly shows distinct differences when the IEF materials are incorporated with both PCBM and C_60_. Samples without the perovskitoid (labeled PCBM and C_60_) exhibit rapid decay dynamics, which are typically reported for these systems (∼40–50 ns effective lifetime). When the perovskitoid is included in the layer stack, however, there is a very clear slowdown in the decay dynamics for all traces, with decay lifetimes in the range of 2–4 μs. In addition, the collected data sets for IEF-PCBM samples exhibit an extremely fast initial decay with an effective lifetime of ∼4 ns, while IEF-C_60_ samples exhibit an initial fast decay that is 1 order of magnitude slower, in the range of ∼100 ns. Full details regarding the lifetimes can be found in Table S1, and the fits to the TRPL data are shown in Figure S12.

**Figure 5:**
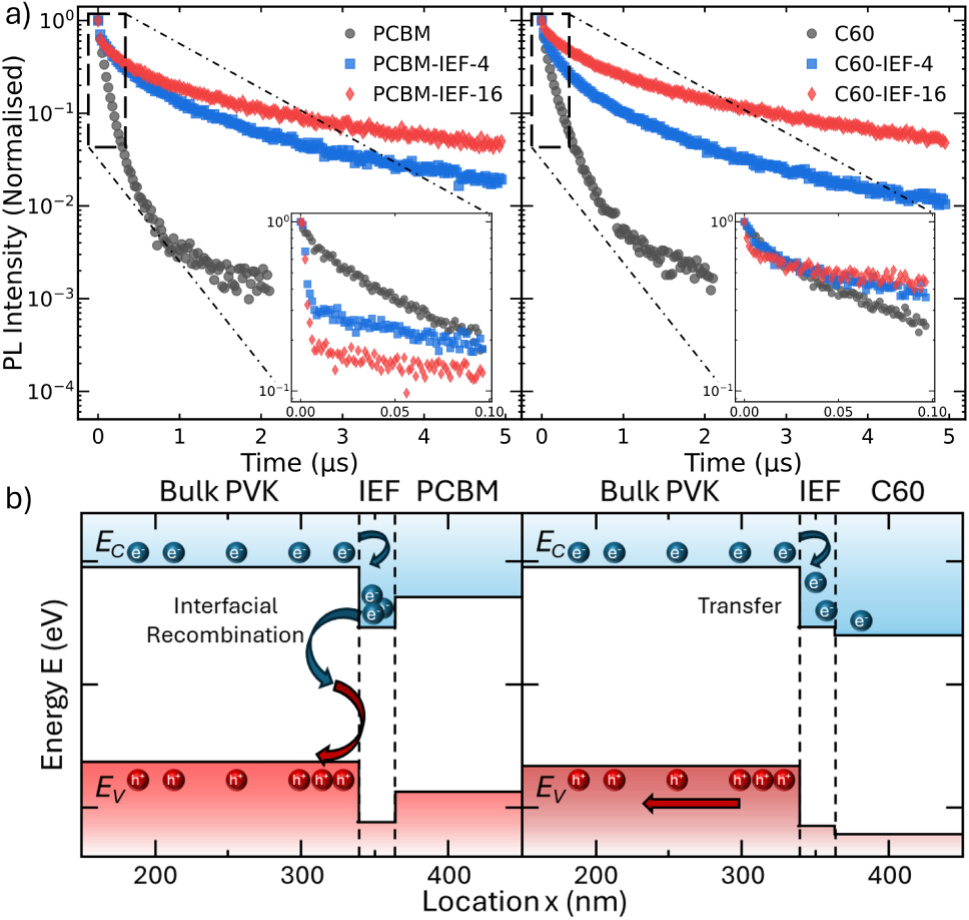
a) Normalized time-resolved photoluminescence (TRPL) decays for samples with the structure Glass|PVK|IEF|ETL. Insets show the early decay dynamics for the first 100 ns. b) Schematic illustration of the impact the energetic mismatch has on charge accumulation and recombination dynamics for both PCBM and C_60_ with the IEF present.

In perovskite/charge extraction bilayer films, the long-term component is typically associated with recombination dynamics, while the early time typically reflects charge transfer into the ETL.^45^ We can therefore understand the TRPL decay as follows for samples including the perovskitoids: efficient electron transfer into the IEF unambiguously accounts for the early time decay fast dynamics (see the insets of TRPL). However, charge transfer from the perovskitoid into the ETL is hindered due to a mismatch between the CB of the IEF and the LUMO of the PCBM and to a lesser extent for C_60_. Electron accumulation in this layer will thus attract holes from the bulk of the perovskite through Coulomb interactions, forcing a large interfacial charge carrier density, which will inevitably lead to increased interfacial recombination and likely accounts for the accelerated initial decay observed in this sample set.^45^ This is schematically illustrated in Figure 5b.

Taken together, the TRPL results provide conclusive evidence that the incorporation of Bi-based perovskitoid interlayers leads to modulation of the conduction band edge at the perovskite/ETL interface. The slower charge extraction dynamics and charge buildup within the IEF layers explain why the solar cells with these interlayers underperform the PCBM control despite the more favorable energetic alignment with C_60_. In particular, the accumulation of carriers in the IEF slows electron transfer and promotes interfacial recombination, which accounts for the observed voltage losses. Nevertheless, the measurements clearly demonstrate that these perovskitoids can act as effective interfacial modifiers, capable of tuning the perovskite conduction band and thereby opening a viable route for fine control of interfacial energetics in perovskite optoelectronic devices.

## 4 Conclusions

In this work, we demonstrate the use of bismuth-based perovskitoids as interlayers to modulate interfacial energetics in perovskite solar cells (PSCs) across both the NIP and PIN device architectures. Here, UPS and XPS studies identified deep conduction and valence bands of the IEF-4 and IEF-16 perovskitoids, which can be employed to selectively induce or minimize energy-level differences across perovskite solar cells. When utilized in NIP architectures, the IEF-16 interfacial layer between the perovskite and Spiro-OMeTAD blocks effective hole extraction, as characterized by S-shaped *J*–*V* curves and a reduction in photovoltaic performance. Similarly, the addition of bismuth perovskitoids in PIN architectures utilizing PCBM as an ETL results in reduced electron extraction. TRPL measurements provide conclusive evidence of this effect: while fast transfer into the perovskitoid is observed, subsequent transfer into PCBM is hindered, resulting in charge accumulation in the IEF, increased interfacial recombination, and associated voltage losses. In contrast, when C60 is used as the ETL, the much better energy level alignment with IEF-4 facilitates more efficient charge transfer, leading to significant improvements in device performance. The PCE increased from 2.29% when only using C_60_ to 14.20% with IEF-4, which we attribute to the minimal 0.1 eV conduction band offset between the two materials and improved charge transfer between the perovskite absorber and the IEF-4 interlayer. Overall, this work demonstrates that bismuth-based perovskitoid materials can effectively control band alignment within perovskite devices. They serve to broaden the energetic landscape of potential charge extraction layers, offering a pathway toward more robust, lead-reduced perovskite optoelectronic devices with enhanced performance.

## Supplementary Material



## Data Availability

All supporting data are available in the Supporting Information.

## Notes

Optimization of the deposition protocol for bismuth-based perovskitoids, collected data of NIP architecture devices, optimization of TC perovskite deposition protocol, PL measurements of ETL depositions on TC/IEF-X samples, optimization of bismuth-based perovskitoids deposition protocol on the perovskite absorber and collected data of PIN architecture devices (PDF)
