# Supplementary material for: Controlling Band-Bending for Perovskite Optoelectronic Devices Using Bismuth-Based Interlayers
Source: ACS Omega. 2025 Oct 27;10(43):52067–75. doi: 10.1021/acsomega.5c09745 (PMC12593018; doi:10.1021/acsomega.5c09745)
Supplement: Supplementary file 1 [file ao5c09745_si_001.pdf]

Controlling band-bending for perovskite optoelectronic devices using bismuth-based interlayers

Rubén Serrano-Nieto,[1,5] Wai Kin Yiu,[2] Marcin Giza,[2] Fraser J. Angus,[2] Graeme Cooke,[2] Patricia Horcajada,[1] Yolanda Pérez,[1,3*] Pablo Docampo[2,4*]

[1]*IMDEA Energy Institute, Advanced Porous Materials Unit (APMU), Avda. Ramón de la Sagra 3, E-28935 Móstoles, Madrid, Spain.*

[2]*School of Chemistry, University of Glasgow, Joseph Black Building, G12 8QQ, Glasgow, Scotland, United Kingdom*

[3]*COMET-NANO group, Departamento de Biología y Geología, Física y Química Inorgánica, ESCET, Universidad Rey Juan Carlos, Calle Tulipán s/n, 28933, Móstoles, Madrid, Spain*

[4]*Basque Centre for Materials, Nanostructures and Applications, 48940, Leioa, Spain*

[5]*Departamento de Química Inorgánica, Facultad de Ciencias Químicas, Universidad Complutense de Madrid, Avenida Complutense s/n, 28040 Madrid, Spain*

Electronic Supporting Information

SI_1. Optimization of the deposition protocol for bismuth-based perovskitoids on glass substrates.

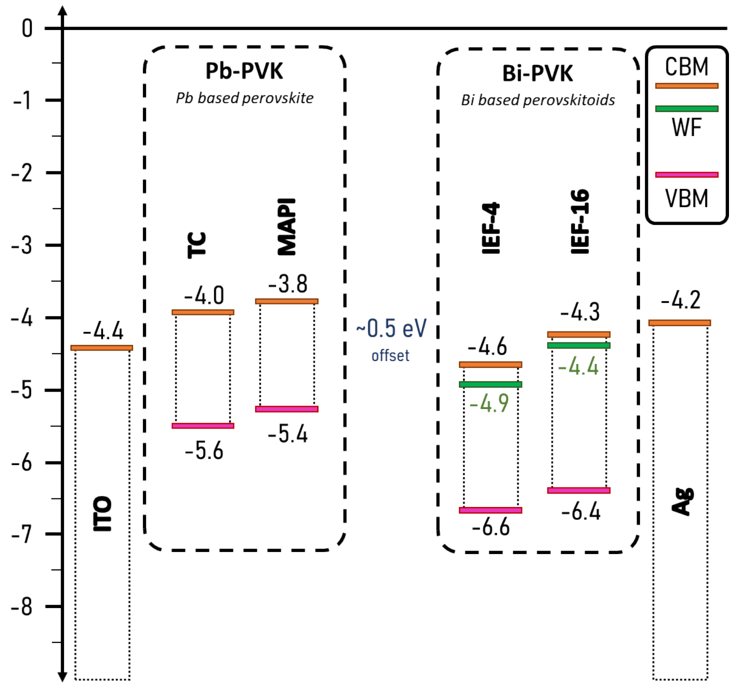

Figure S1. Energy scheme of energy levels (valence band minimum, VBM; conduction band minimum, CBM; and work function, WF) of IEF-4 and IEF-16 bismuth-based perovskitoids compared to lead-based perovskites.

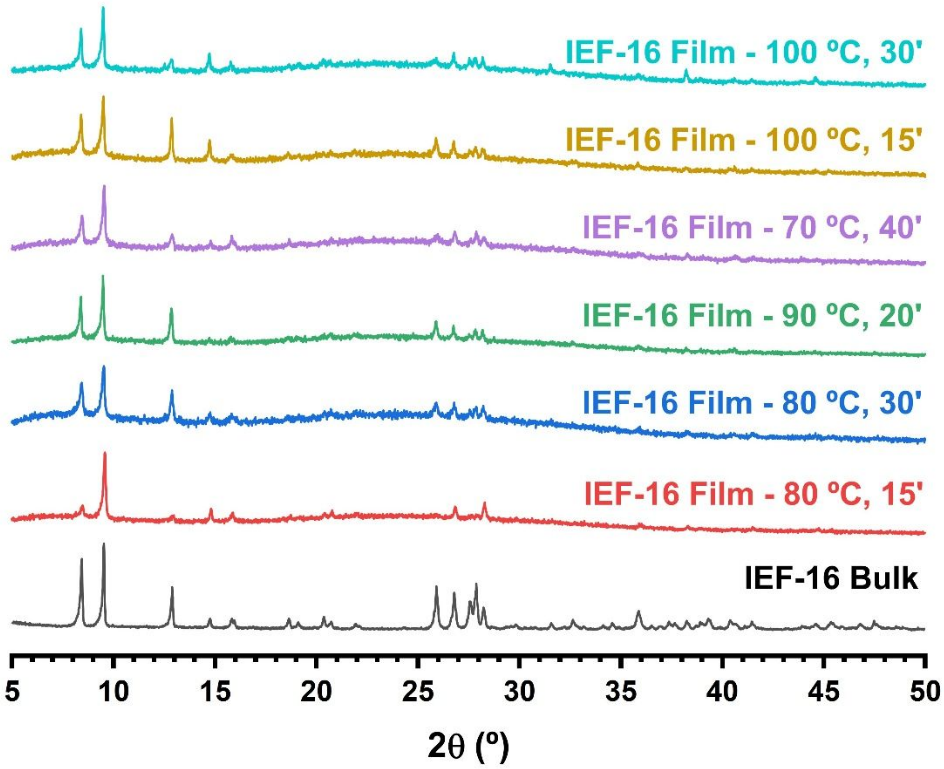

Figure S2. XRD diffractograms of IEF-16 powder (black) and IEF-16 films at different annealing conditions.

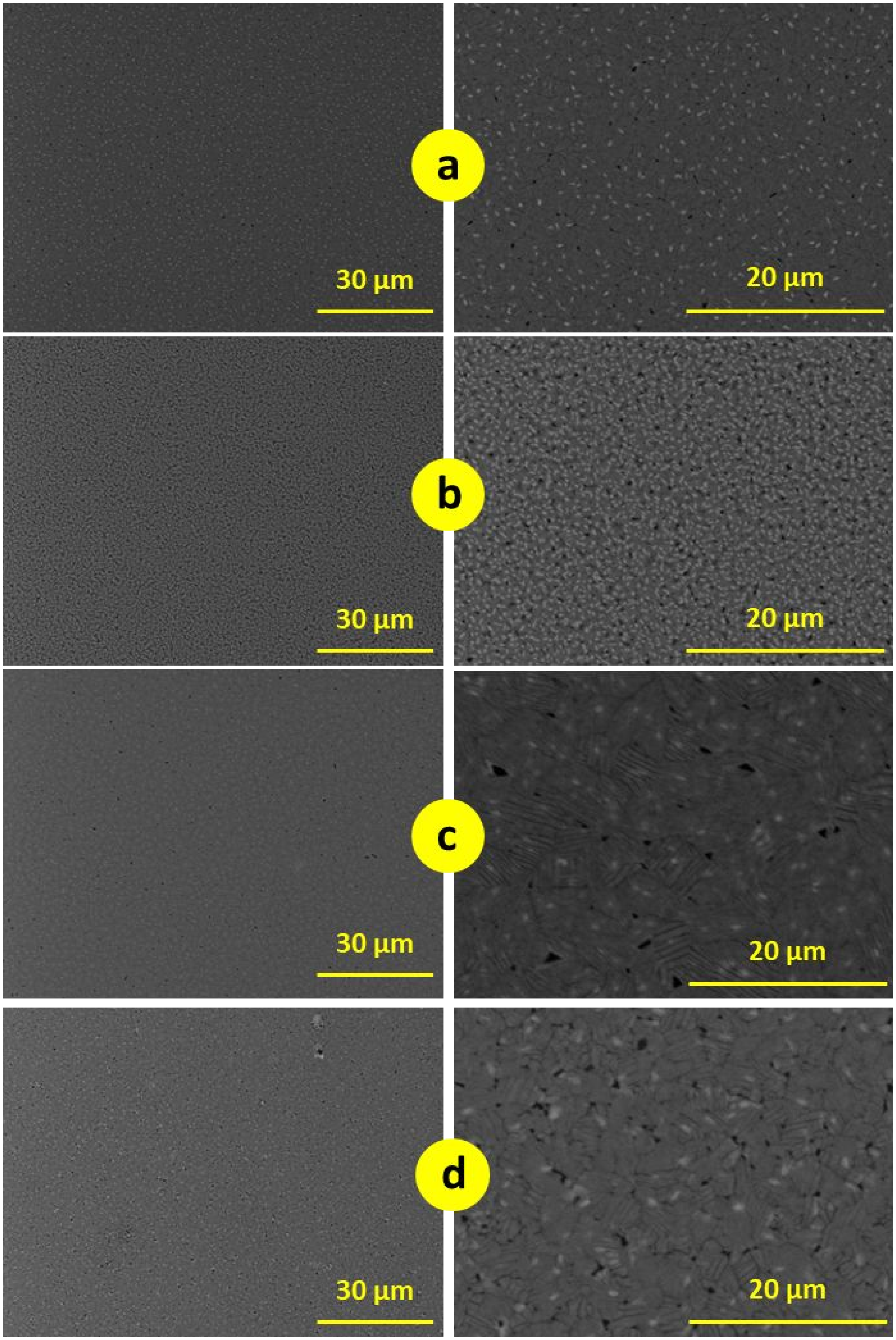

Figure S3. SEM images of IEF-16 films formed at different annealing conditions: a) 80 °C for 15 min; b) 70 °C for 40 min; c) 50 °C for 60 min; and d) 100 °C for 15 min.

SI_2. Collected data of NIP architecture devices using bismuth-based perovskitoids

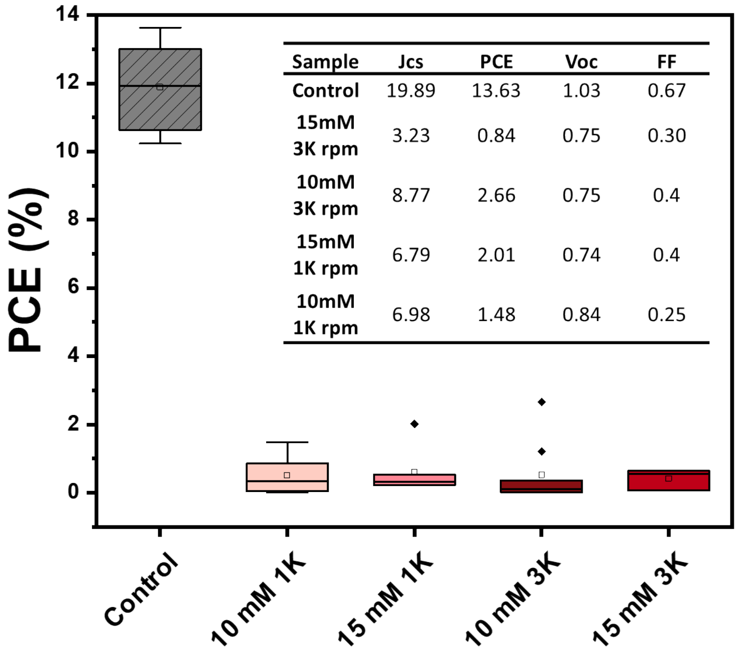

Sample	Jcs	PCE	Voc	FF
Control	19.89	13.63	1.03	0.67
15mM 3K rpm	3.23	0.84	0.75	0.30
10mM 3K rpm	8.77	2.66	0.75	0.4
15mM 1K rpm	6.79	2.01	0.74	0.4
10mM 1K rpm	6.98	1.48	0.84	0.25

Figure S4. Box plot of PCE values and (inset) table of J_{sc}, FF, and V_{oc} values for FTO/SnO_2/MAPI/IEF-16/Spiro/Au architecture exploring different concentrations and spin-coating protocols of IEF-16.

SI_3. Optimization of TC perovskite deposition protocol on glass substrates

Triple-cation perovskites (TCs), composed of FA^+/MA^+ mixed ions, incorporating cesium (Cs) as a cation, with chloride or bromide anions, have demonstrated superior thermal and structural stability.[1,2] Based on the prior experience within our group, the TC perovskite system was selected as the absorber layer for further optimization in PIN architectures. The deposition process of TC perovskites was optimized through spin-coating tests to achieve the highest crystallinity and uniform film formation. Three different deposition protocols were used (**Figure S5**), each varying in the TC precursor solution deposition timing at the first step (static or dynamic at 5 or 15 s), as well as the volume of ethyl acetate (EA) used (100, 200 or 300 µL) as the anti-solvent, which is added during the second step of the spin-coating process at different times (15, 25 or 35 s). The static deposition of the TC, followed by the addition of 200 µL of EA at 15 s during the second step, and annealing at 100 °C for 30 min, produced the most uniform films with minimal defects (**Figure S5**), as evidenced by the smooth surfaces and the well-crystalline XRD (**Figure S6**). Thus, this protocol of deposition of the TC was selected for device fabrication.

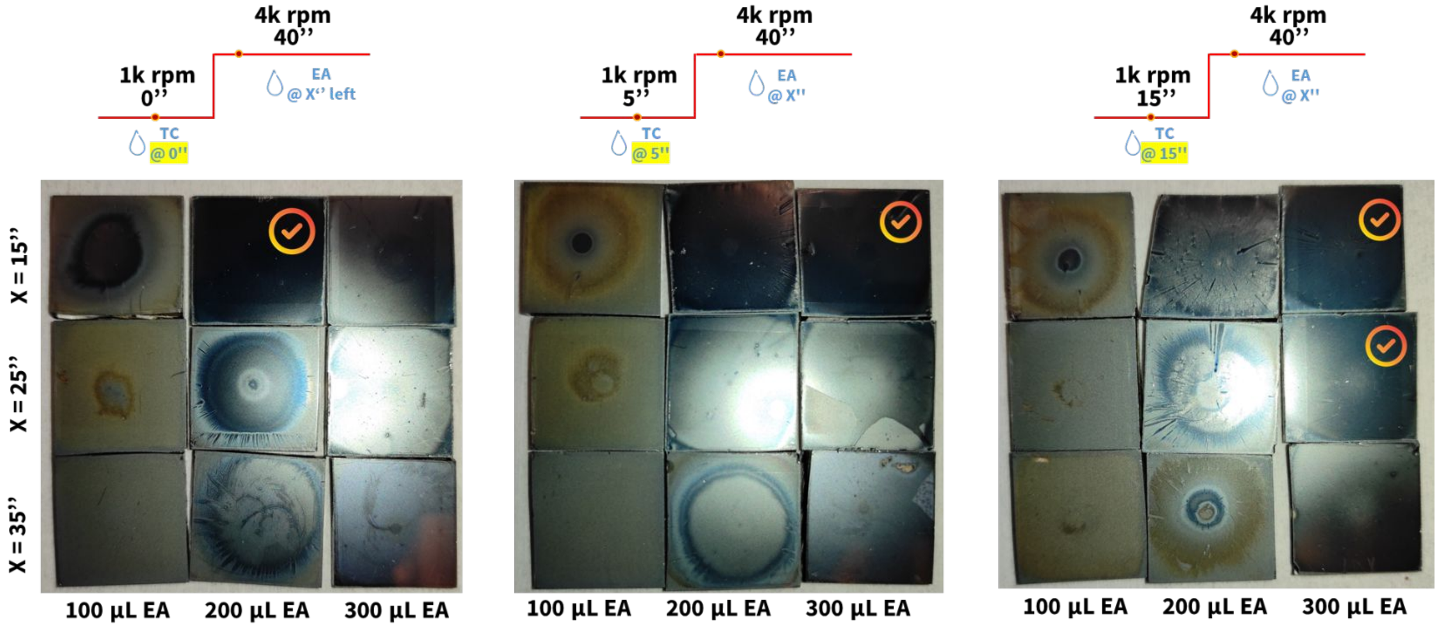

Figure S5. Images of optimization of the deposition of the TC.

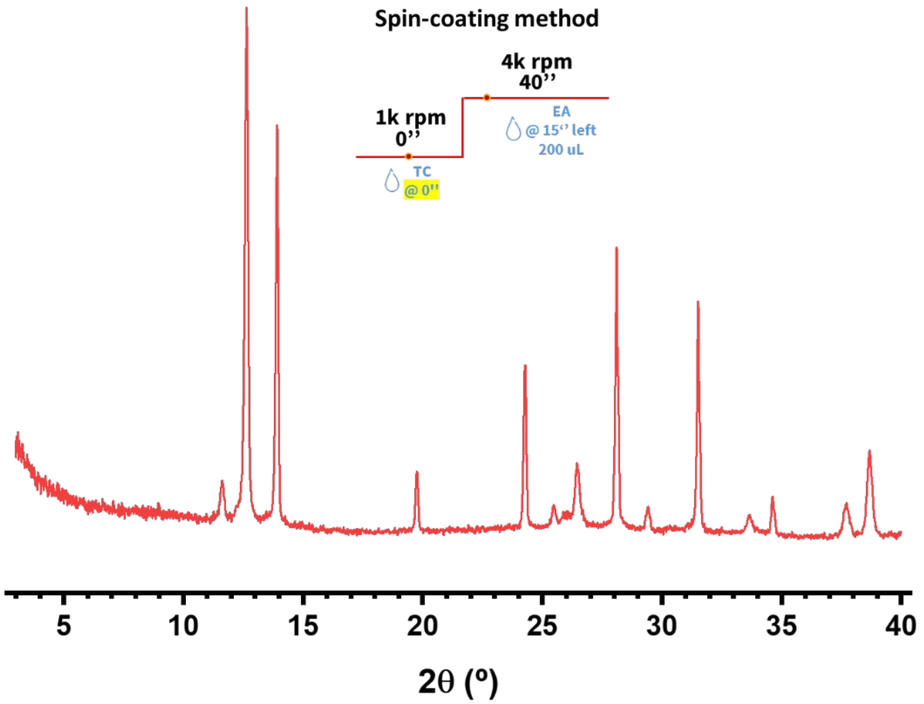

Figure S6. XRD pattern of the best TC film after deposition optimization.

SI_4. PL measurements of ETL depositions on TC/IEF-X samples.

Photoluminescence (PL) spectroscopy was carried out on a series of samples with architecture Glass/TC/IEF-X/ETL to evaluate the effect of different interfacial combinations on charge carrier recombination. Here, TC corresponds to the lead halide perovskite absorber, IEF-X refers to IEF-4 or IEF-16 (bismuth-based perovskitoids), and ETL denotes C_{60} or PCBM. The ETL layers were prepared from solutions of 10 mg·mL^{-1} in solvents, deposited dynamically at 3000 rpm for 30 s, and annealed at 70 °C (5 min) and 100 °C (15 min) for C_{60} and PCBM, respectively. The PL spectra (**Figure S7**) reveal that including IEF-X consistently suppresses radiative recombination compared to the reference samples. This effect is observed for both ETLs but is more pronounced when PCBM is used, likely due to its less optimal energy level alignment with the lead perovskite. In contrast, the PL intensity is already lower in C_{60}-based samples, indicating a more efficient baseline extraction of electrons and suggesting better band alignment between C_{60} and the TC layer.

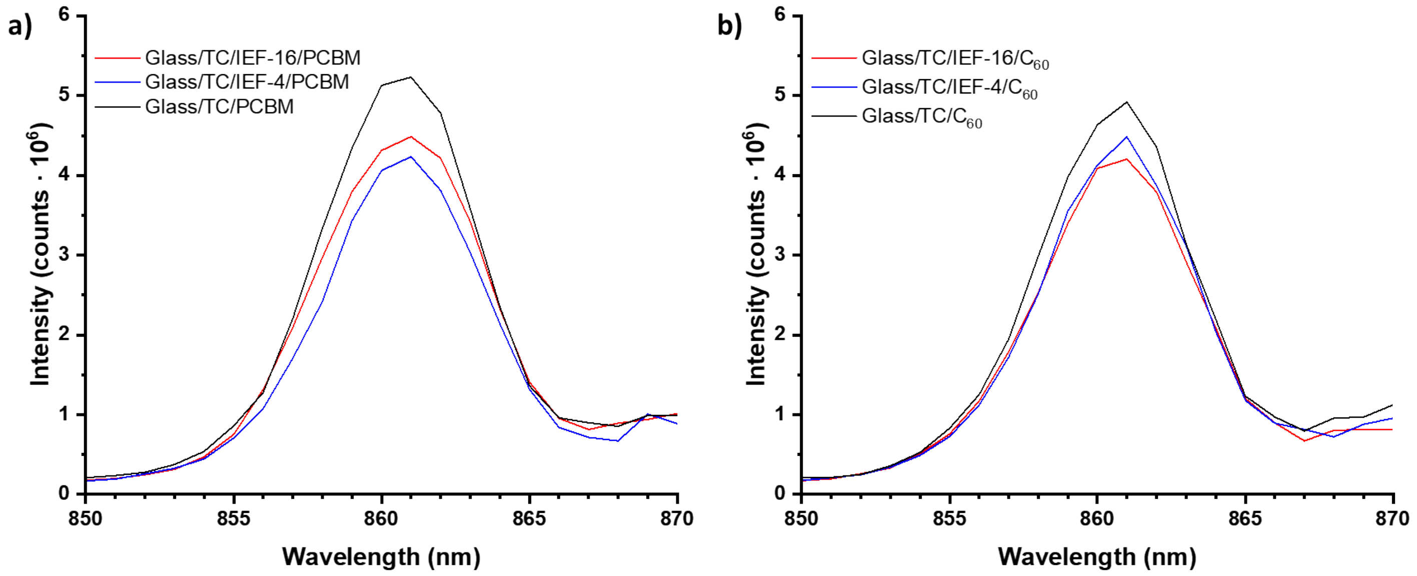

Figure S7. PL measurements of the TC absorber using (a, b) PCBM and C_{60} as ETLs (black) before and (red, blue) after IEF-16 and IEF-4 Bi-based perovskitoids deposition as interlayers.

SI_5. Optimization of bismuth-based perovskitoids deposition protocol on glass substrates.

The optimization of adding Bi-based materials as interlayers focused on achieving uniform film deposition, optimal crystallization, and an intimate contact interface with the perovskite absorber. The process of IEF-16/TC formation was fine-tuned by systematically adjusting three key parameters: solution concentration (1.25, 2.5, or 5 mM), and annealing conditions (50 °C for 60 min or 100 °C for 15 min). Due to the lower concentration of IEF-16 (1.25-5 mM) compared to the TC perovskite concentration (3.12 M), the signals corresponding to the formation of the IEF-16 film were not observed in the XRD analysis (**Figure S8)**, only providing clear evidence of the formation of the TC layer.

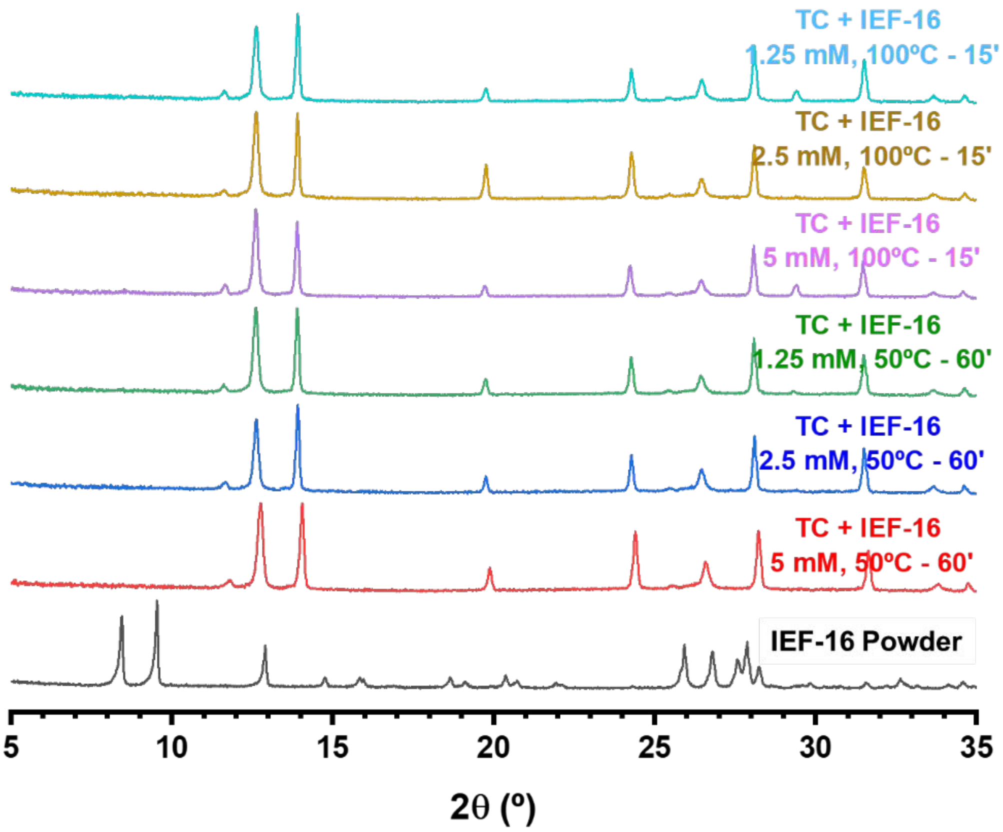

Figure S8. XRD patterns of the IEF-16 powder and the resulting films containing IEF-16 deposited at different concentrations and annealing conditions onto the TC layer.

SI_6. Collected data of PIN architecture devices using bismuth-based perovskitoids

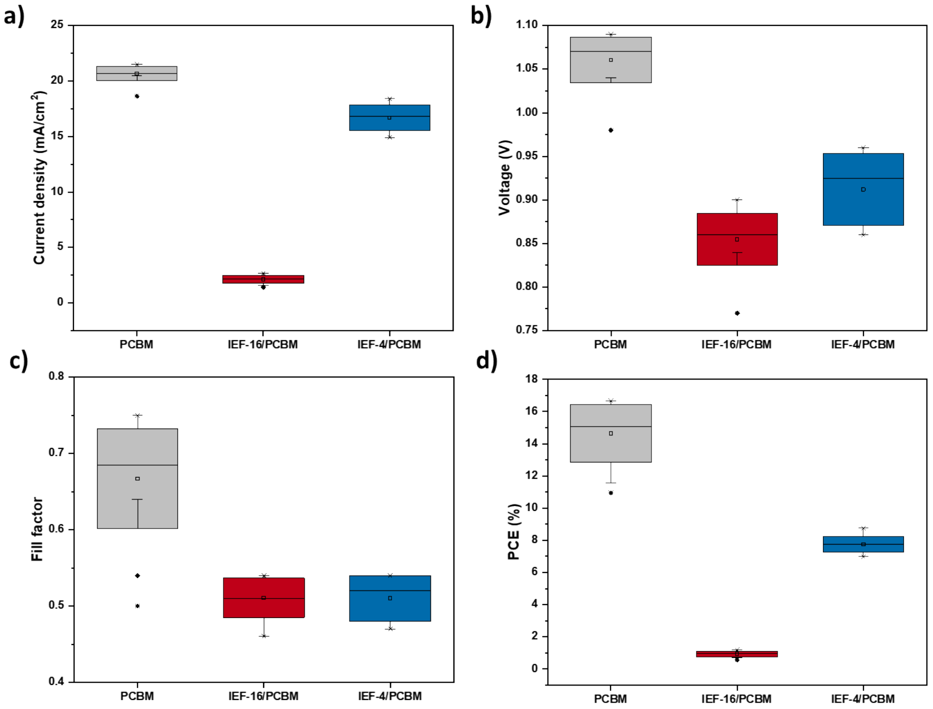

Figure S9. Performance parameters of ITO/MeO-2PACz/Al_2O_3-NPs/TC/IEF-X/PCBM/BCP/Ag architecture using IEF-16 (red) and IEF-4 (blue) bismuth-based perovskitoids under simulated solar illumination for forward scan; a) J_{sc}, b) V_{oc}, c) FF, and d) PCE. (8 devices for each condition)

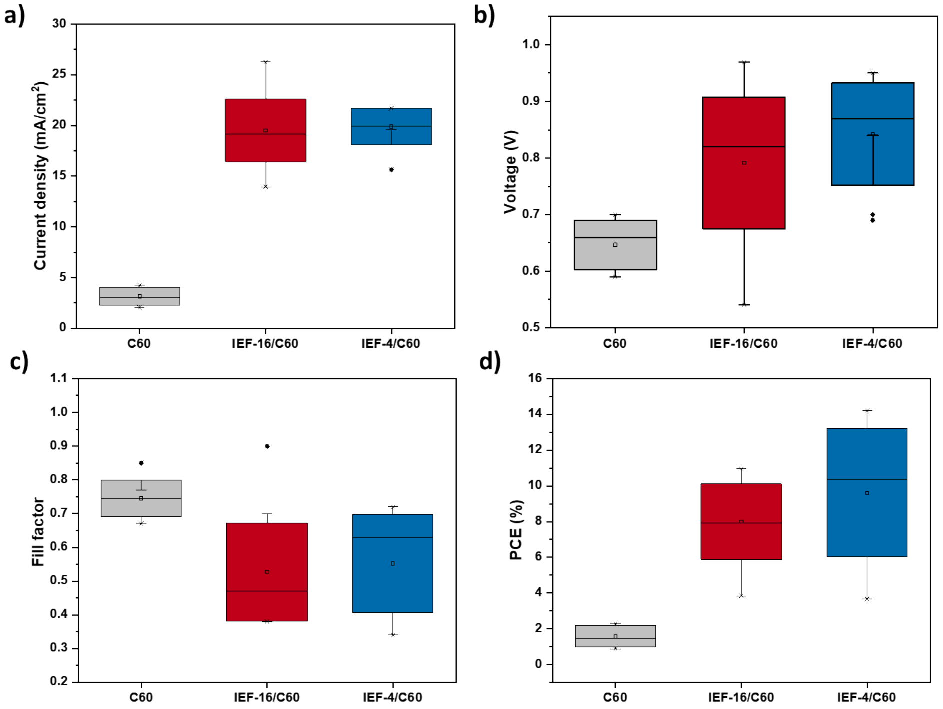

Figure S10. Performance parameters of ITO/MeO-2PACz/Al_2O_3-NPs/TC/IEF-X/PCBM/C_{60}/Ag architecture using IEF-16 (red) and IEF-4 (blue) bismuth-based perovskitoids under simulated solar illumination for forward scan; a) J_{sc}, b) V_{oc}, c) FF, and d) PCE (8 devices for each condition)

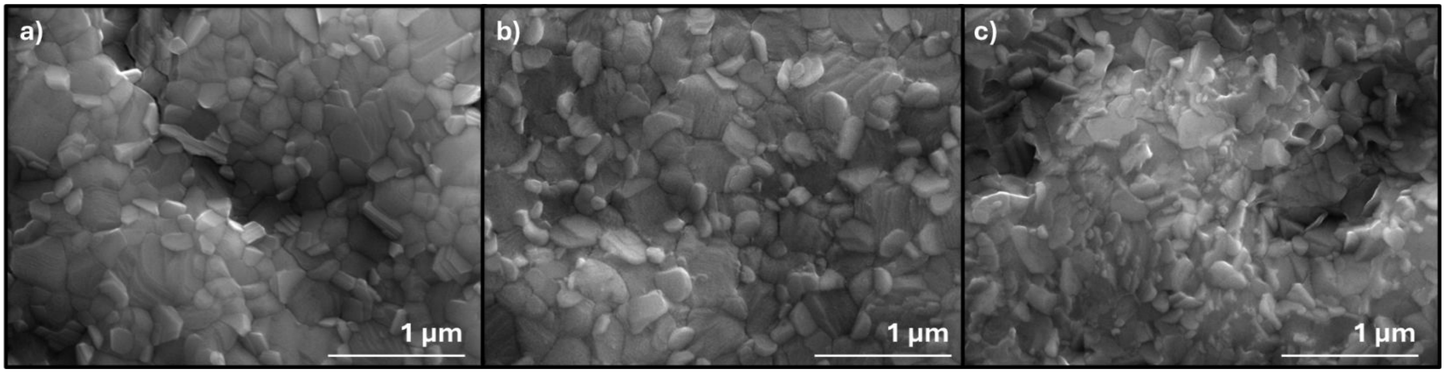

Figure S11. SEM images of a) Triple cation perovskite; b) Triple cation perovskite coated with 3.6 mM of IEF-4; c) Triple cation perovskite coated with 10.8 mM of IEF-16. All samples were deposited on ITO substrates, following spin coating protocols for fabricating inverted solar cells.

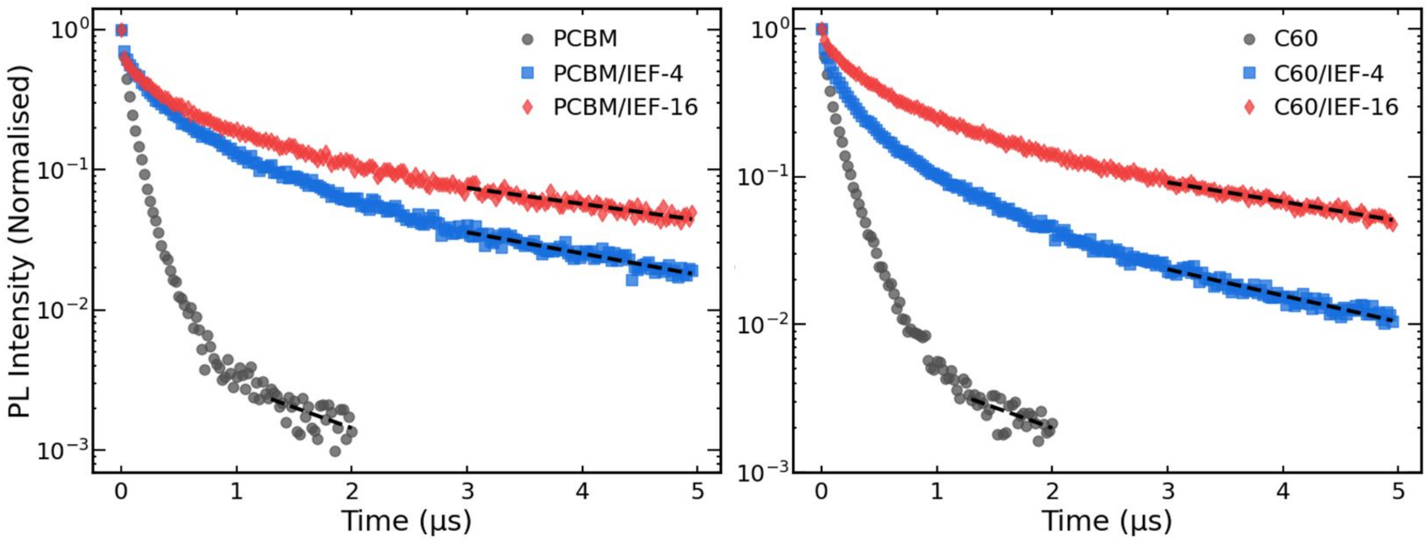

Figure S12. TRPL Decays with black dashed lines showing the mono-exponential fit for the lifetime extraction.

Table S1. Lifetimes of all samples extracted from the TRPL using a mono-exponential fit, $(I(t) = I_0 e^{-t/\tau})^3$ to the tail of the decay and effective lifetime (1/e).

Sample	Lifetime (τ, from fit) (ns)	Effective Lifetime (τ_1/e) (ns)
**C_{60}**	1563.6	49.9
**C_{60}|IEF-4**	2437.9	82.9
**C_{60}|IEF-16**	3324.4	159.9
**PCBM**	1471.9	44.9
**PCBM|IEF-4**	2864.3	5.2
**PCBM|IEF-16**	3780.7	3.5

REFERENCES

(1) Su, J.; Liang, S.; Zhao, Z.; Yu, T.; Zou, S.; Jiang, Y.; Liang, C.; Zhang, M.; Chen, W.; Shi, L.; Guo, Y.; Yu, Y.; Dong, Y. Components and Defect Density Optimization of FAxMA1-xPbI3 Based on Simulation for High Performance Perovskite Solar Cells. *Current Applied Physics* **2024**, *67*, 38–45. https://doi.org/10.1016/j.cap.2024.07.013.

(2) Hu, Z.; An, Q.; Xiang, H.; Aigouy, L.; Sun, B.; Vaynzof, Y.; Chen, Z. Enhancing the Efficiency and Stability of Triple-Cation Perovskite Solar Cells by Eliminating Excess PbI2 from the Perovskite/Hole Transport Layer Interface. *ACS Appl. Mater. Interfaces* **2020**, *12* (49), 54824–54832. https://doi.org/10.1021/acsami.0c17258

(3) Al-Ashouri, A. *et al.* Monolithic perovskite/silicon tandem solar cell with >29% efficiency by enhanced hole extraction. *Science* **2020**, *370*, 1300-1309. https://doi.org/10.1126/science.abd4016.
